# Evidence for Positive Selection on the *Osteogenin* (*BMP3*) Gene in Human Populations

**DOI:** 10.1371/journal.pone.0010959

**Published:** 2010-06-04

**Authors:** Dong-Dong Wu, Wei Jin, Xiao-Dan Hao, Nelson Leung Sang Tang, Ya-Ping Zhang

**Affiliations:** 1 State Key Laboratory of Genetic Resources and Evolution, Kunming Institute of Zoology, Chinese Academy of Sciences, Kunming, China; 2 Laboratory for Conservation and Utilization of Bio-resource, Yunnan University, Kunming, China; 3 Graduate School of the Chinese Academy of Sciences, Beijing, China; 4 KIZ/CUHK Joint Laboratory of Bioresources and Molecular Research in Common Diseases, Kunming, China; 5 Department of Chemical Pathology, Faculty of Medicine, The Chinese University of Hong Kong, Hong Kong, Special Administrative Region, People's Republic of China; 6 Laboratory for Genetics of Disease Susceptibility, Li Ka Shing Institute of Health Sciences, The Chinese University of Hong Kong, Hong Kong, Special Administrative Region, People's Republic of China; University of Illinois at Champaign-Urbana, United States of America

## Abstract

**Background:**

Human skeletal system has evolved rapidly since the dispersal of modern humans from Africa, potentially driven by selection and adaptation. Osteogenin (BMP3) plays an important role in skeletal development and bone osteogenesis as an antagonist of the osteogenic bone morphogenetic proteins, and negatively regulates bone mineral density.

**Methodology/Principal Findings:**

Here, we resequenced the *BMP3* gene from individuals in four geographically separated modern human populations. Features supportive of positive selection in the *BMP3* gene were found including the presence of an excess of nonsynonymous mutations in modern humans, and a significantly lower genetic diversity that deviates from neutrality. The prevalent haplotypes of the first exon region in Europeans demonstrated features of long-range haplotype homogeneity. In contrast with findings in European, the derived allele SNP Arg192Gln shows higher extended haplotype homozygosity in East Asian. The worldwide allele frequency distribution of SNP shows not only a high-derived allele frequency in Asians, but also in Americans, which is suggestive of functional adaptation.

**Conclusions/Significance:**

In conclusion, we provide evidence for recent positive selection operating upon a crucial gene in skeletal development, which may provide new insight into the evolution of the skeletal system and bone development.

## Introduction

Humans are characterized by dramatic skeletal anatomic differences from the chimpanzee, our closest neighbor in evolution. For example, bipedalism, walking and running upright on two feet, is evident in many earliest hominins [1], however, human bipedalism is much more energy economical than in other apes, and requires some specialized adaptations of the skeleton and muscles [2]. These changes in skeleton and muscles likely have a genetic basis and were accompanied by changes in selective pressures. Modern human populations also demonstrate substantial phenotypic diversity, which can be illustrated by differences in body mass, height, and craniofacial dimensions that are all shaped by the skeletal system. The rapid evolution of the skeletal system during modern human evolution has previously been reviewed [3]. Therefore genes that regulate bone development are good potential targets for positive selection, and the identification of genes that were the subject of adaptive evolution should be a good strategy to investigate the mechanisms of gene evolution in skeletal development, and associated diseases. Indeed, a flood of genes have been reported as undergoing positive selection and responsible for the adaptive features that are observed in humans, such as genes associated skin pigment [4], [5], [6], [7], [8], lactase persistence in response to dairy farming [9], [10], [11], *EDAR* and *EDA2R* involved in hair morphology [7], [12], human brain development genes [13], [14], as well as many others (reviewed in [15], [16]). Recently, we concluded that skeletal genes demonstrated high population differentiation driven by positive selection, supporting the rapid evolution of skeletal system [17].

Bone morphogenetic proteins (BMPs) are members of the transforming growth factor-beta (TGF-beta) superfamily, and play important roles in skeletal development and osteogenesis by regulating cell type specification and cell differentiation [18]. BMP3, also known as osteogenin, is an antagonist for several of the osteogenic BMPs, and is a negative determinant of bone density [19]. For example, *BMP3* (−/−) mice have twice as much trabecular bone as wild-type littermates [19]. Therefore, BMP3 should be a good candidate to investigate for evidence of positive selection on skeletal genes during human evolution.

Here, we re-sequenced the *BMP3* gene in 145 modern human individuals from four different populations. We show that positive selection has operated on *BMP3* as evidenced by several population genetics signals, i.e. greater numbers of non-synonymous mutations, a lower genetic diversity, and longer-range haplotype homozygosity. Our findings support the hypothesis that there was a period of rapid evolution on at least one of the osteogenic genes during human evolution.

## Results

A total of 290 chromosomes from unrelated human individuals were investigated by sequencing, covering three exonic regions of the *BMP3* locus. Furthermore, we also analyzed the evolutionary patterns by employing the genotyped data for the *BMP3* gene from HapMap [20].

To detect evidence of positive selection we utilized five different population genetics methods that analyze variation in DNA sequence for signatures of selection. These signatures include: 1) high proportion of function altering mutations; 2) reduction in genetic diversity; 3) high-frequency of derived alleles; 4) high genetic differentiation between populations; 5) long range haplotypes and strong linkage disequilibrium [16]. We examined the sequences for these signatures of *BMP3* variation in human populations for evidence of positive selection operating on *BMP3*.

### Excessive non-synonymous mutations

**Figure 1:**
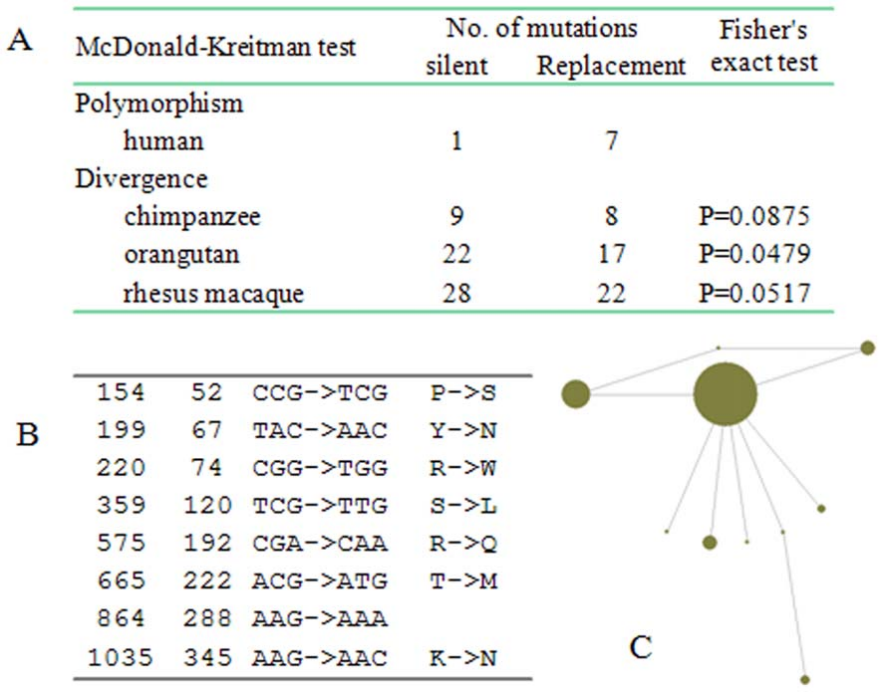
Summary of the polymorphisms in the *BMP3* coding region. (A) Summary of the McDonald-Kreitman test; (B) represents positions of the mutations in the mRNA, protein, codon and amino acid changes from left to right; and (C) indicates the network of the haplotypes constructed from the eight SNPs.

Under neutral evolution, the ratio of non-synonymous (replacement) to synonymous (silent) substitutions is expected to be constant within and between species, but the value will deviate if positive selection is operating, providing the rationale for the McDonald-Kreitman test [21]. Total 11 mutations were found in the sequenced region. We compared the polymorphism data at coding sequence of *BMP3* among human populations, with the divergence between human and other primates, i.e. chimpanzee, orangutan, rhesus macaque. Among the 8 mutations found in the coding sequence, 7 are nonsynonymous and 1 is synonymous (Fig. 1A and 1B). The cross species comparison comes to the conclusion that there are more nonsynonymous SNPs within human population than expected (the difference is statistically significant with orangutan, *P* = 0.0479, and are marginally significant with chimpanzee, *P* = 0.0875, and rhesus macaque, *P* = 0.0517) (Fig. 1). Although positive selection could increase the numbers of nonsynonymous mutations, such deviation from neutrality could also be attributable to a recent relaxation of the selective constraint in the human population. We considered that relaxation of selection as being improbably, because of the low Ka/Ks values (e.g. 0.304 between human and chimpanzee, 0.231 between human and rhesus macaque, and 0.128, the average value among mammals) were indicative of strong purifying selection operating on the *BMP3* throughout mammalian evolution, and the known fact that *BMP3* plays an essential role in skeletal system [19]. The pattern of substitutions is suggestive that *BMP3* gene probably evolved in a non-neutral manner, possibly driven by positive natural selection, in modern human populations. The phylogenetic network of the haplotypes constructed from these 8 coding sequence mutations showed a star-like pattern, consistent with a history of positive selection (Fig. 1C).

### Lower level of nucleotide diversity

The first exon of the *BMP3* gene (583 bp in length) has 7 haplotypes, and likewise for exon 2 (955 bp). In contrast, no sequence variation was observed in the coding region of exon 3. The nucleotide diversity, π, defined as the average number of pairwise nucleotide sequence difference, is 3.02×10^−4^ for the complete sequenced region, a value significantly lower than the empirical data of the SeattleSNP genes (95.8% percentile rank) (http://pga.gs.washington.edu/). This low value is a signature of positive selection, most likely due to a selective sweep caused by genetic hitchhiking [16]. Furthermore, a series of classical tests, i.e. Tajima's D [22], Fu and Li's D, F, D*, F* [23], [24], were employed to detect deviation from neutrality (table 1). Positive values for these parameters indicate evidence of balancing selection or of a population subdivision event; in contrast, negative values indicate purifying selection, positive selection, or may be the result of demographic history, e.g. population expansion. In the whole human panel, the first exon showed significantly negative Fu and Li's F and F* values (*P*<0.05, table 1). To exclude the confounding effect of demographic history, a coalescent simulation was constructed incorporating the best-fit demographic parameters for each population [25] to calculate Tajima's D, Fu and Li's D, F, D*, and F*. After controlling for demographic history, only the exon 1 region continues to demonstrate a significant negative Fu and Li's D* value for the East Asian population (*P*<0.05, table 1). These negative values can not be attributable to purifying selection as there is an excess of non-synonymous mutations, thus we conclude that exon 1 region was largely involved in positive selection in the East Asian population. No alleles were found with a high derived allele frequency, and as expected, Fay and Wu's H [26] testing for derived alleles did not yield a significant result. Fu's Fs statistic that compares the observed number of haplotypes with that expected from neutrality and showed many significant signals, which indicated a pattern of haplotype diversity that deviated from the expectation under neutrality. However, Fu's Fs method is particularly sensitive to demographic effects, and therefore, we can not conclude whether positive selection or demographic history (e.g. population expansion) account for the observed pattern.

**Table 1:** Summary statistics of the *BMP3* gene in human populations.

	Nucleotide Diversity	Tajima's D	Fu and Li's D	Fu and Li's F	Fu and Li's D*	Fu and Li's F*	Fay and Wu's H	Fu's Fs
African (N = 66)								
Total (1951 bp)	0.000581379	−0.844	0.534	0.083	0.532	0.090	1.007	**−4.093**
Exon1 region (583 bp)	0.000657873	−0.827	−0.508	−0.711	−0.486	−0.690	0.324	−1.399
Exon2 region (955 bp)	0.000808408	−0.608	1.086	0.639	1.072	0.630	0.683	**−2.565**
European (N = 72)								
Total	0.000180077	−0.826	−0.528	−0.727	0.508	−0.709	0.308	**−2.712**
Exon1 region	0.000463705	−0.566	−1.011	−1.024	−0.991	−1.005	0.231	−0.650
Exon2 region	8.48021E−05	−0.747	0.510	0.164	0.514	0.165	0.077	−0.748
East Asian (N = 78)								
Total	0.000148324	−0.464	−1.030	−1.003	−1.011	−0.985	0.241	−0.486
Exon1 region	4.39794E−05	−1.055	−1.982	−1.988	*−1.966*	−1.971	0.025	−2.009
Exon2 region	0.000276168	0.362	0.505	0.538	0.509	0.541	0.216	0.911
South Asian (N = 74)								
Total	0.000265484	−0.772	−0.213	−0.459	−0.196	−0.443	0.406	−1.583
Exon1 region	0.00018163	−1.211	−1.018	−1.257	−0.998	−1.238	0.102	−2.348
Exon2 region	0.000431487	0.007	0.708	0.579	0.710	0.579	0.304	0.186
Whole populations (N = 290)								
Total	0.000301763	−1.524	−0.963	−1.412	−0.947	−1.397	0.548	**−11.296**
Exon1 region	0.00033282	−1.546	−2.206	**−2.365**	−2.187	**−2.349**	0.187	**−7.017**
Exon2 region	0.000413301	−0.926	0.954	0.369	0.952	0.368	0.360	**−3.735**

Bold numbers indicate statistical significance at the 95% level as detected by the DnaSP program. The italic number indicates significance at the 95% level by coalescent simulation incorporating the best-fit demographic parameters.

### Long range haplotype homozygosity

**Figure 2:**
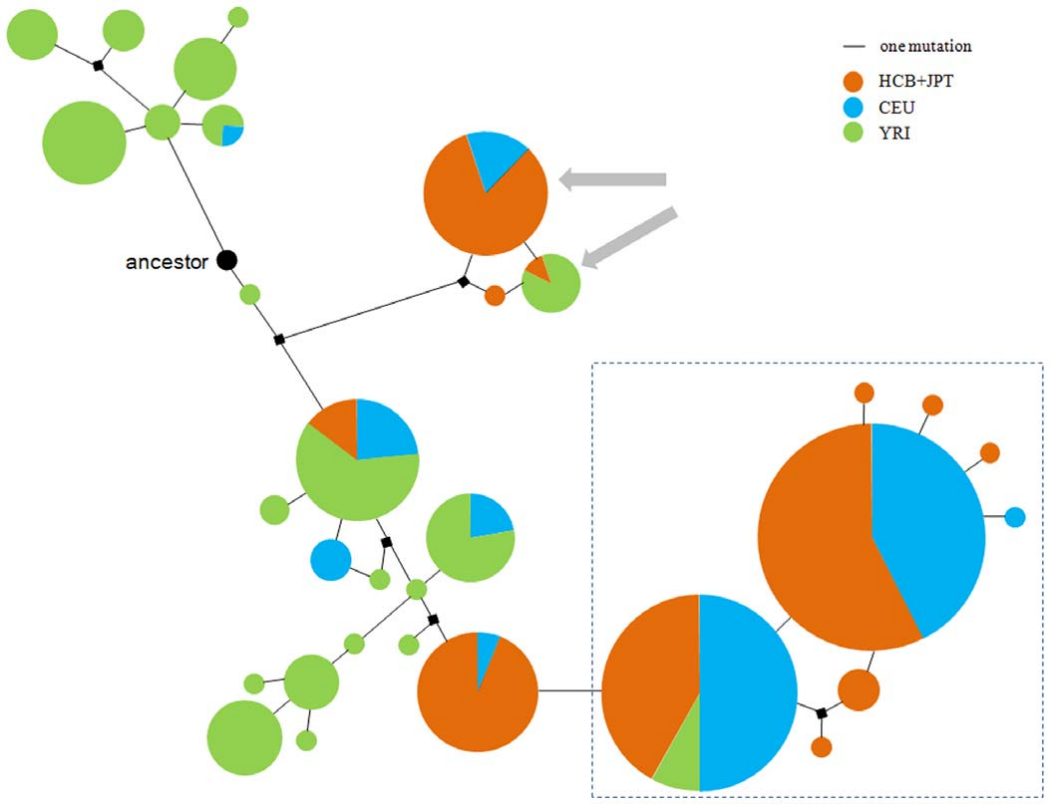
Median-joining (MJ) network constructed by 32 distinct haplotypes inferred from 40 SNPs within the *BMP3* gene. Each haplotype is represented by a circle with its area proportional to its frequency. The black node represents the ancestral haplotype which carries the ancestral allele at each position. The squares represent non-sampled haplotypes inferred by the MJ method. The arrows indicate two haplotypes carrying the derived allele of rs3733549 (Gln), which may be the target of selection in the East Asian population. The dashed squares label the dominant haplotypes under positive selection in the European population.

**Figure 3:**
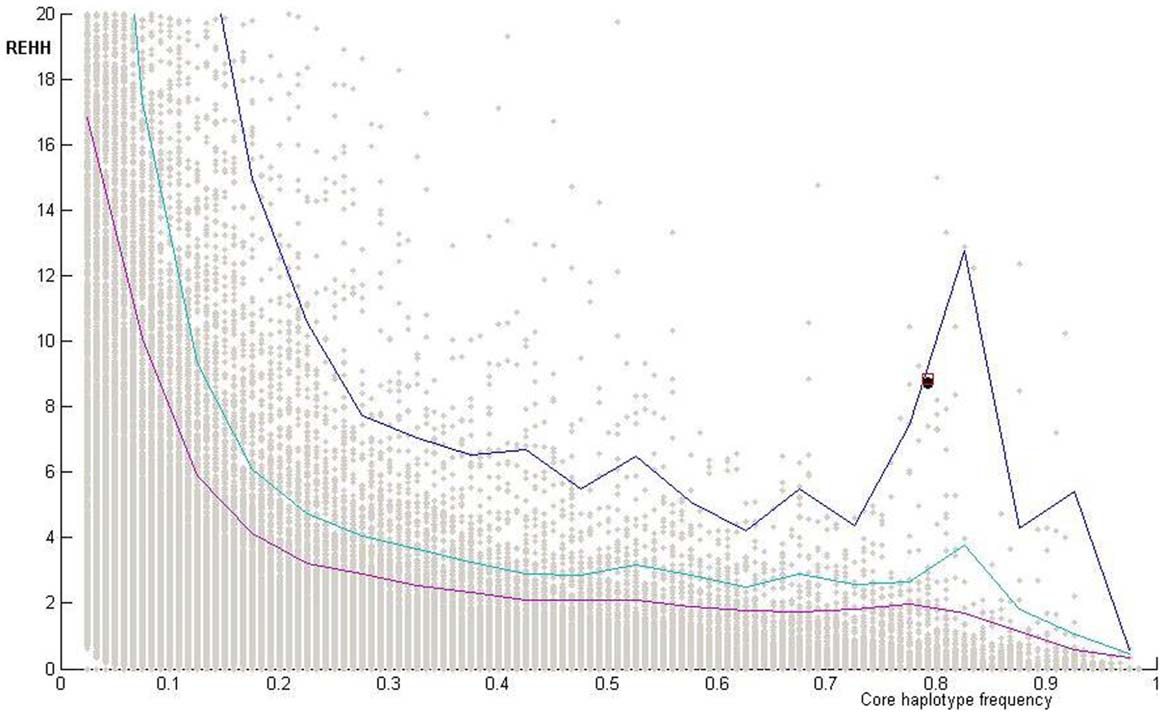
REHH versus Core haplotype frequency of chromosome 4 in CEU populations. REHH was calculated at ∼300 kbp both upstream and downstream of each core haplotype. The 99^th^, 95^th^, and 90^th^ percentiles are drawn.

To further explore this potential target of selection, a median-joining (MJ) network [27] was constructed to describe the evolutionary relationships between the 32 haplotypes, which were inferred from the 40 SNPs in the HapMap project (Phase II, release 21) within the *BMP3* gene regions (Fig. 2). The low levels of reticulation reflect that gene conversion plays a minor role in the population variation of *BMP3* in human populations and is attributable to high LD. There is a high degree of divergence of haplotype composition between African and Non-Africans. Two major clades account for ∼78.3% haplotypes for CEU, and these are also the major haplotypes in East Asians (HCB+JPT) but are rare (only ∼5.3%) in Africans (YRI). The presence of a common high frequency haplotypes in Non-Africans is suggestive of a potential positive selective sweep driving the increase of haplotype frequency. To test this hypothesis, we applied the long-range haplotype (LRH) test, which is based on the feature that a variant underwent recent positive selection and will have a substantial effect on the surrounding region and result in a long range linkage disequilibrium where mutation and recombination have had insufficient time to decay the LD [28]. We retrieved the phased haplotype data for a 1 M bp region that spans the *BMP3* gene from HapMap Phase II data (release 21) [20] that are polymorphic in the three individual populations: African (YRI), European (CEU) and East Asian (CHB+JPT), for analysis of the haplotype homozygosity pattern. The entire chromosome 4, where the *BMP3* gene is located, was used to generate an empirical distribution for the calculation of the statistical *P*-value. This approach is robust to the confounding effects of demography. We defined the core haplotypes using 11 SNPs within the *BMP3* gene region that are within the same block. The LRH statistic, REHH (relative extended haplotype homozygosity), of the prevalent haplotype with frequency 79.2%, reach 8.83, and 8.74 at ∼300 kb upstream and downstream of the core region, respectively (at the 99.61% and 99.57% percentiles rank, respectively at the frequency bin: 0.75–0.80) (Fig. 3). These observations are indicative of positive selection driving the prevalence of the haplotype in Europeans with empirical P values<0.01. Intriguingly, the dominant haplotype forms the major clades in CEU (marked by dashed squares in Fig. 2). In contrast, the core region shows no significant high REHH value in East Asian (HCB+JPT) or African (YRI) populations. Another method, iHS (integrated haplotype score), a modification of the EHH statistic [29], also found a signal of recent positive selection in the CEU population, and identified two SNPs with significant higher iHS values (i.e. rs1106107, rs1994990).

**Figure 4:**
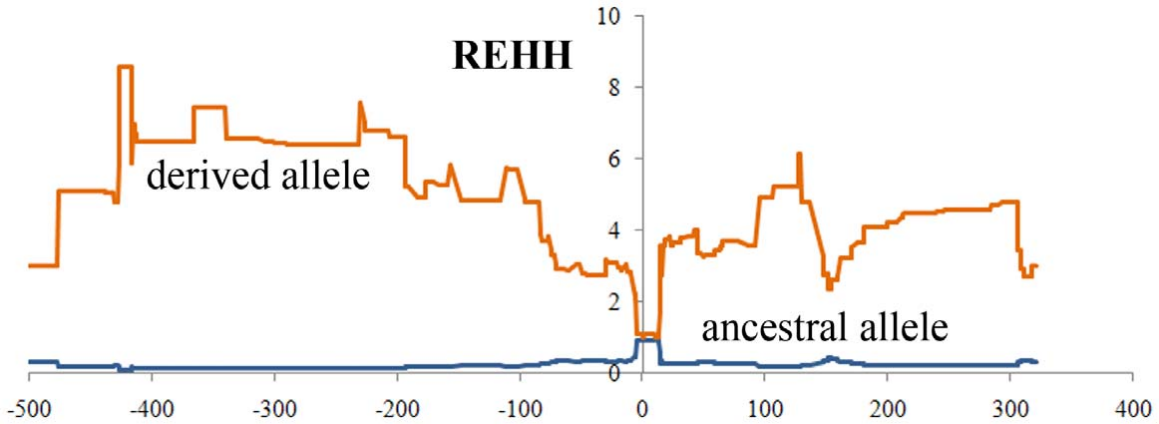
REHH of the derived and ancestral alleles of SNP rs3733549 at varying physical distances (kb).

**Figure 5:**
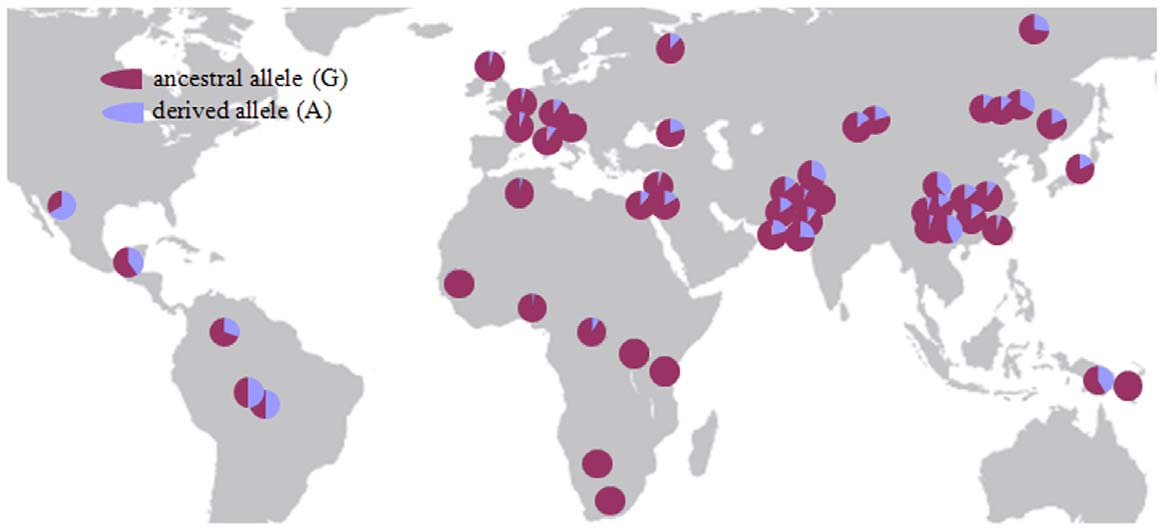
Worldwide distribution of the allele frequency of the SNP rs3733549 (Arg192Gln) genotyped in this study.

Among the seven nonsynonymous mutations, one variant, Arg192Gln, is mutated from a basic amino acid to an acidic amino acid, and is present at a relatively high derived allele frequency in Asian populations (15.38% in East Asian, and 21.97% in South Asian). A change of an amino property may potentially be important in functional innovation and adaptation. In contrast, the Arg192Gln allele frequency is lower in the other populations (4.17% in European, and 4.55% in African). The Arg192Gln mutation has been genotyped by HapMap (rs3733549 (G/A)), which also confirms our findings (with derived allele (A) frequency 15.91% in East Asian, 5% in European, and 5.8% in African). In the phylogenetic network, the only two haplotypes containing the derived allele of rs3733549 demonstrated an unusual pattern of divergence from others (marked by arrows in Fig. 2). The long-range haplotype test based on the HapMap data indicate that in the East Asian population the derived allele demonstrated a higher REHH value than the ancestral allele (Fig. 4), which is a signal of recent positive selection. In East Asians, we suggest that BMP3*192Gln is the potential target of selection, however we still do not know what the functional change is between the two variants, a question that need further examination (e.g., the association with bone system parameters in the East Asian population). To better understand the evolution of *BMP3*, we analyzed the world-wide distribution of the SNPs (Fig. 5) and confirmed that the derived allele was at a relatively high frequency in Asians. Interestingly, the derived allele also demonstrates a higher frequency in five American regions, which suggests that the positive selection force may also be in operation in these regions.

## Discussion

Humans have adapted to the striking differences and changes in environmental features (e.g. diet, climate, and pathogen load) since migrating out of Africa. The human skeletal system has evolved rapidly, particularly since the advent of agriculture, which shapes the phenotypic diversity observed within modern humans [3]. For example, histomorphometric analysis has indicated that there was a greater bone remodeling rate in the agriculturalists than in the hunter-gatherers [3]. In addition, modern humans demonstrate high phenotypic variation among different populations, which to a large extent can be illustrated by skeletal differences, such as height, bone mineral density, skull diversity, and craniofacial dimensions. Therefore, skeletal system is a likely a target of selection during human evolution. Actually, our recent study concluded that skeletal genes showed high population differentiation among humans driven by positive selection [17]. Previous studies have indicated that many positively selected genes are responsible for human adaptive features (reviewed in [16]).

In this study, although many potential factors, such as, purifying selection, mutation rate, population demographic events, and genetic drift, confound our observation, the simplest explanation for the observed deviation from neutrality (i.e., greater numbers of nonsynonymous mutations, lower genetic diversity, strong linkage disequilibrium, and long-range haplotype homozygosity) of *BMP3* in humans is the operation of positive natural selection. The concordant presence of multiple signatures (detected by population genetic testing) of positive selection on a single region of the human genome is unlikely due to the different mechanisms. For example, previous genome-wide scans for positive selection in humans based on different mechanisms or statistical tests revealed a low level of concordance among the identified positively selected genes [16]. Here, we provide evidence of positive selection on *BMP3* which is supported by several different signals including the excess of non-synonymous mutations, lower genetic diversity, and long-range haplotype homozygosity in modern human populations. The selection that operated at *BMP3* probably was maintained for a long period of time, and may still be ongoing, since the different methods have differing sensitivity in the detection of selection at different time scales. For example, the statistics based on haplotype homozygosity are better at identifying an incomplete selective sweep which suggests a very recent event [16]. In contrast, methods based on the differing proportions of non-synonymous and synonymous mutations is considered to be best at detecting selection that acted over a much longer time scale [16].

The adaptive evolution of *BMP3* is consistent with the rapid evolution of the human skeletal system, although we do not have data that explains the mechanism for the selective advantage of the *BMP3* variant. BMP3, is an antagonist of several osteogenic BMPs, and is a negative determinant of bone density [19]. Lacking the BMP3, mice have increased bone mass [19]. Potentially, the antagonistic activity of human BMP3 to osteogenic acting factors, and even the level of BMP signal, was adaptively changed via many amino acid substitutions during human evolution, which may diverge functionally from chimpanzee accounting for the skeletal differences [2], [30]. It still needs test by further functional experiment. The targets of selection operated on the *BMP3* are different between European and East Asian evidenced by long-range haplotype test (Fig. 2). Within modern human populations, *BMP3* may also diverge in the activity, expression level, accounting for the skeletal variation, such as body mass, because of the key function of BMP3 in the skeletal system [19], [31], [32].

In conclusion, we show strong evidence for positive selection operating on the *BMP3*, a gene which plays an important role in skeletal development and bone osteogenesis. These findings should provide valuable information on the evolution of the human skeletal system and bone development, and it is likely that other osteogenic genes have also experienced similar evolutionary pressures and are good candidates for future molecular evolutionary studies.

## Materials and Methods

### Sequencing of *BMP3* gene

Since the three coding exons of the *BMP3* gene are separated by two long introns we only sequenced the three coding regions, including some flanking sequences, in individuals from four human populations: 33 Africans, 36 Europeans, 39 East Asians and 37 South Asians. All individuals were unrelated and chosen randomly from the Human Genome Diversity Cell Line Panel [33]. DNA sequencing was performed on an ABI 3730 automated DNA sequencer. Primer and PCR condition are available on request. Sequences were analyzed by DNAStar software. The sequences of each individual were submitted to GenBank under accession numbers GQ291587-GQ292021.

The genotypes of non-synonymous SNP rs3733549 in the unrelated individuals in the world-wide panel of Human Genome Diversity Cell Line Panel [33] were obtained by an independent PCR and cut with the diagnostic restriction enzyme Taq I, and separated on an agarose gel.

### Statistics of population genetics

Haplotypes were inferred by the PHASE program [34], [35] based on the mutations discovered by sequencing. Tajima's D [22], Fu and Li's D, F, D*, F*, Fs [23], [24], [36], and Fay and Wu's H [26], were employed to detect a deviation from neutrality by the program DnaSP 4.0 [37]. Tajima's D is a commonly used summary of the site-frequency spectrum of nucleotide polymorphism data, and is based on the difference between two estimators of θ (the population mutation rate 4*Ne*µ): nucleotide diversity that is the average number of pairwise differences between sequences, and Watterson's estimator [38], based on the number of segregating sites. A negative Tajima's D signifies an excess of low frequency polymorphisms, and indicates a population size expansion and/or positive selection, or negative selection. A positive Tajima's D value indicates a decrease in population size and/or that balancing selection is operating [15]. Methods for Fu and Li's parameters are similar, and are also based on the difference between estimators of θ. To exclude the confounding effect of demographic history, a coalescent simulation was constructed incorporating the best-fit demographic parameters for each populations [25], and Tajima's D, Fu and Li's D, F, D*, and F*, were calculated by the PopGen package in the bioperl [39]. Fay and Wu's H is based on the level of SNPs with high derived allele frequencies. Here, the derived allele of each SNP was deduced based on the chimpanzee and orangutan sequences from UCSC (http://genome.ucsc.edu/).

The median-joining (MJ) network [27] was constructed to describe the evolutionary relationships between the 32 haplotypes, inferred from the genotypes of 40 SNPs in the HapMap project PHASE II (release 21). The phased haplotype data from a 1 M bp region that spans the *BMP3* gene from HapMap that are polymorphic in the three individual populations: African (YRI), European (CEU) and East Asian (CHB+JPT), was used to construct the long-range haplotype test by analyzing the extended haplotype homozygosity pattern. The entire data from chromosome 4, where *BMP3* is located, were used as an empirical distribution to calculate the statistical *P*-value.
